# From discovery to application: twenty years of *Meyerozyma caribbica*

**DOI:** 10.1093/femsyr/foag010

**Published:** 2026-02-11

**Authors:** Elisângela de Souza Miranda Muynarsk, Angela Alves dos Santos, Cristina Link Rüntzel, Brigitte Sthepani Orozco Colonia, Rafaela de Oliveira Penha, Bárbara Braga Vieira Marques, Danilo Grunig Humberto da Silva, Sergio Luiz Alves, Jr

**Affiliations:** Campus of Três Lagoas, Federal University of Mato Grosso do Sul, 79613-000 Três Lagoas, MS, Brazil; Institute of Biosciences, Humanities and Exact Sciences, Bioscience Postgraduate Program, São Paulo State University (UNESP), 15054-000 São José do Rio Preto, SP, Brazil; Postgraduate Program in Biotechnology and Biosciences, Federal University of Santa Catarina, 88035-972 Florianópolis, SC, Brazil; Laboratory of Yeast Biochemistry, Federal University of Fronteira Sul, 89815-899 Chapecó, SC, Brazil; Department of Biochemistry, Federal University of Santa Catarina, 88035-972 Florianópolis, SC, Brazil; Bioprocess Engineering and Biotechnology Department, Federal University of Paraná, Centro Politécnico, 81531-990 Curitiba, PR, Brazil; Bioprocess Engineering and Biotechnology Department, Federal University of Paraná, Centro Politécnico, 81531-990 Curitiba, PR, Brazil; Institute of Biosciences, Humanities and Exact Sciences, Bioscience Postgraduate Program, São Paulo State University (UNESP), 15054-000 São José do Rio Preto, SP, Brazil; Campus of Três Lagoas, Federal University of Mato Grosso do Sul, 79613-000 Três Lagoas, MS, Brazil; Institute of Biosciences, Humanities and Exact Sciences, Bioscience Postgraduate Program, São Paulo State University (UNESP), 15054-000 São José do Rio Preto, SP, Brazil; Postgraduate Program in Biotechnology and Biosciences, Federal University of Santa Catarina, 88035-972 Florianópolis, SC, Brazil; Laboratory of Yeast Biochemistry, Federal University of Fronteira Sul, 89815-899 Chapecó, SC, Brazil

**Keywords:** biodegradation, non-conventional yeasts, biocontrol, probiotics, xylitol

## Abstract

Over the past two decades, *Meyerozyma caribbica* has been identified as a metabolically versatile and ecologically adaptable yeast with significant relevance to biotechnology, agriculture, environmental remediation, and food applications. Since its formal description in 2005, this species has demonstrated the ability to grow on a wide range of substrates and under various stress conditions, facilitating the production of valuable bioproducts such as ethanol, xylitol, arabitol, and volatile aroma compounds. Multiple strains efficiently ferment lignocellulosic hydrolysates, tolerate inhibitory compounds, and remain active at elevated temperatures, which supports their application in integrated biorefineries. In addition to its fermentative capabilities, *M. caribbica* serves as an effective biocontrol agent through the production of antifungal metabolites, hydrolytic enzymes, mycoparasitism, nutrient competition, and the induction of plant defense responses. Environmental functions include the degradation of dyes, hydrocarbons, and organochlorine pesticides, as well as metal biosorption and the mitigation of oxidative stress in plants. There is also increasing interest in its potential as a probiotic and as a starter culture that can modulate sensory attributes in fermented foods. This review synthesizes 20 years of research on *M. caribbica*, focusing on its roles in bioproduct production, plant disease management, bioremediation, and probiotic or food-related applications.

## Teaser

This review presents the first comprehensive overview of the yeast *Meyerozyma caribbica*, revealing its growing importance and diverse potential uses in biotechnology, agriculture, and environmental applications.

## Introduction

The microbial conversion of renewable raw materials into biofuels and biotechnologically relevant compounds is essential to advancing a sustainable bioeconomy. As fermenting micro-organisms, yeasts have been used for centuries in traditional fermentation processes, especially in the production of beverages, food, and biofuels (Maicas 2020). The conventional yeast *Saccharomyces cerevisiae*, although the most widely used in industrial fermentation processes, has limitations, such as difficulty in metabolizing complex substrates and sensitivity to stressful environments, which limit its performance in certain applications. In contrast, many other yeast species (called non-conventional yeasts) have demonstrated attributes such as metabolic versatility and physiological resistance, making them promising candidates to drive the next generation of industrial bioprocesses (Geijer et al. 2022).

**Figure 1:**
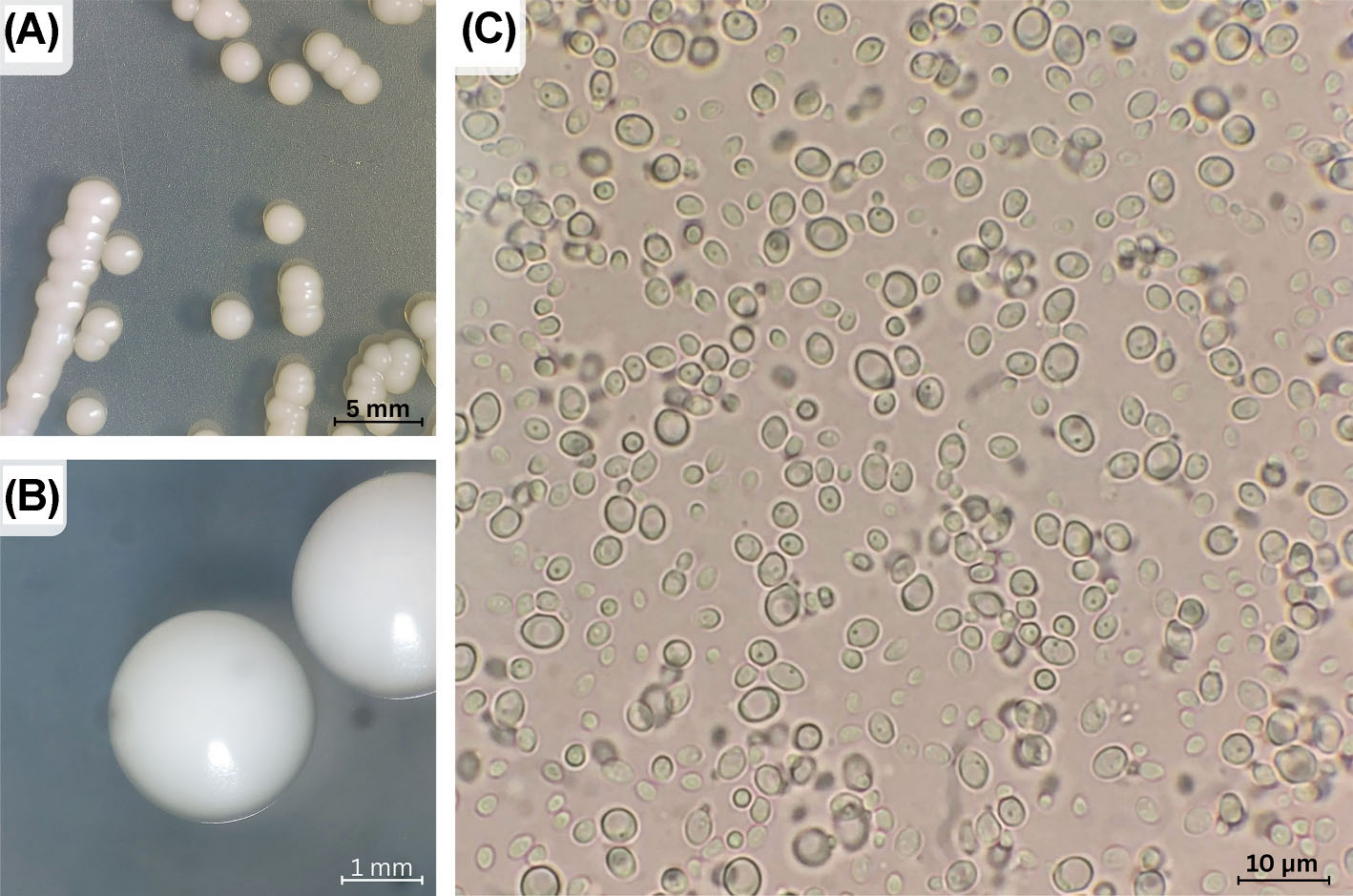
Colony (A, B) and cell morphology (C) of *M. caribbica*, observed by stereomicroscopy and optical microscopy, respectively. Cells were cultivated in solid YPD medium (1% yeast extract, 2% peptone, 2% glucose, and 2% agar) prior to imaging. Photographs were taken by the authors. The strain used was CHAP-282, as described by Giehl et al. (2025).

Among non-conventional yeasts, the species *Meyerozyma caribbica* has stood out in different biotechnological contexts. It has ovoid or elongate cells that reproduce by budding and form small, white, creamy colonies (Fig. 1), which are typical features of its genus (Romi et al. 2014, Pooja et al. 2021). Although originally described as *Pichia caribbica* (type strain DBVPG 4519, NRRL Y-27274, CBS 9966—Vaughan-Martini et al. 2005), it was later reclassified into the new genus *Meyerozyma* based on phylogenetic analyses, particularly through sequencing specific regions of ribosomal DNA (Kurtzman and Suzuki 2010).

*Meyerozyma caribbica* belongs to the *Meyerozyma guilliermondii* complex, which is formed by multiple yeast species that include *M. guilliermondii, M. caribbica, M. athensensis, M. carpophila, M. elateridarum, M. neustonensis*, and *M. smithsonii* (de Marco et al. 2018, Ghasemi et al. 2022). Among these species, *M. caribbica, M. guilliermondii*, and *M. carpophila* are reported to be quite similar, both morphologically and at the genome level, making it difficult to distinguish between them (Romi et al. 2014, de Marco et al. 2018). Despite these high similarities, *M. caribbica* has been identified through several molecular techniques, such as sequencing and analysis of specific regions of ribosomal DNA: the Internal Transcribed Spacer (ITS1-5.8S-ITS2) region (between the 18S and 26S rRNA genes) and the variable domains D1/D2 of the 26S rRNA gene (Romi et al. 2014, Albarello et al. 2023, Agarbati et al. 2024, Fenner et al. 2025). Furthermore, Romi et al. (2014) developed a technique (called internal transcribed spacer restriction fingerprinting) that is able to differentiate *M. caribbica* and *M. guilliermondii* by means of restriction fragments of polymorphisms in the ITS region of yeasts.

**Figure 2:**
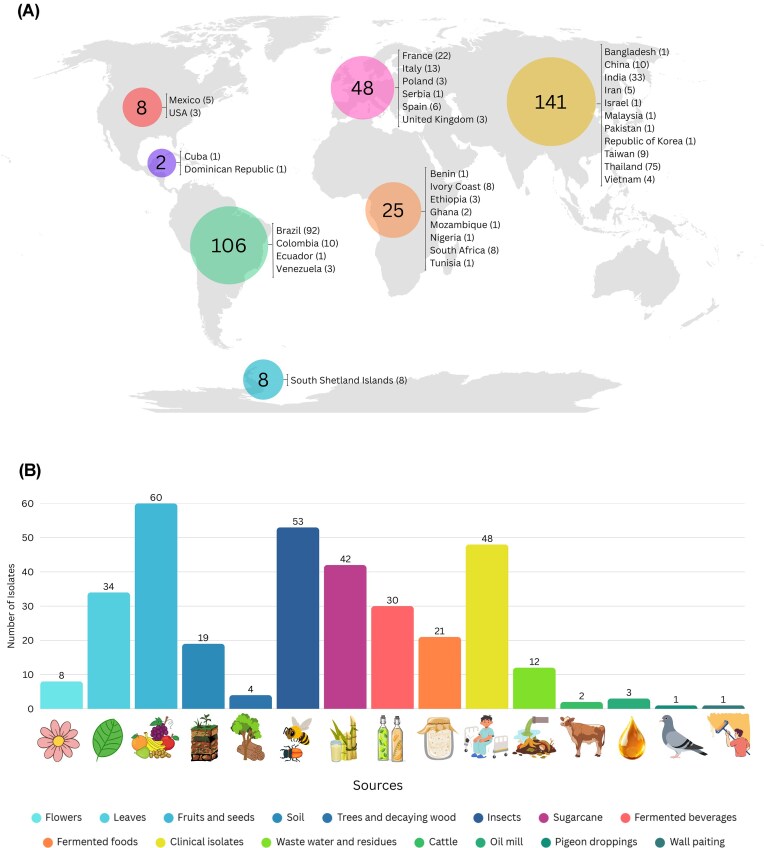
Global distribution of the 338 *M. caribbica* isolates found in the literature, based on a dataset consisting of 232 references retrieved from Scopus using the following search query: TITLE-ABS-KEY (*caribbica*) AND [LIMIT-TO (DOCTYPE, “ar”)]. In A: Geographic distribution of *M. caribbica* isolates reported by continents. Each of the colored circles shows the total number of isolates documented for the respective region. Country-level counts are shown to the right of each circle. In B: Diversity of environmental, food, and clinical sources from which *M. caribbica* strains have been isolated. To construct the figure, we used the references cited in Table S1 of the supplementary material.

Twenty years ago, this species was first described in an article published in this journal (Vaughan-Martini et al. 2005). Over these two decades, several strains of *M. caribbica* have been isolated from a wide variety of environments and substrates around the world, as summarized in Fig. 2 and Table S1. The biochemical characterization of *M. caribbica* isolates has proven useful for important biotechnological processes. Here, we present an overview of this species’ potential in bioproduct generation, plant disease management, bioremediation, and probiotic or food-related applications.

## The potential of *M. caribbica* in generating biotechnological products

### Ethanol, xylitol, and arabitol from renewable raw materials

When exploring strategies to decarbonize the transport sector, bioethanol has been highlighted as an alternative to fossil fuels and as a means to promote the circular economy (Naik et al. 2010, Ahmed and Sarkar 2018, Pooja et al. 2021). In this area, the strain *M. caribbica* MJTm3 has been tested for ethanol production from sugarcane molasses (one of the raw materials for first-generation ethanol—E1G). In a bioreactor under optimized fermentation conditions, the strain produced 49 g/l of ethanol with a productivity of 0.68 g/l/h, representing 92% of the theoretical yield (Hawaz et al. 2023). However, it is important to note that sugarcane and corn used in the production of E1G are food sources and therefore compete directly with the food sector. To overcome this dichotomy, one approach is to utilize the chemical composition of lignocellulosic residues (sugarcane bagasse, wheat straw, wheat bran, corn fiber) to produce so-called second-generation ethanol (E2G) (Alfenore and Molina-Jouve 2016, Ahmed and Sarkar 2018, Moremi et al. 2020, Hawaz et al. 2022).

On the other hand, it is known that E2G production is linked to numerous challenges, since lignocellulosic residues are rich in the polysaccharides cellulose and hemicellulose, which are encased in lignin. Consequently, pretreatment and hydrolysis steps, which precede fermentation, are necessary to make available the fermentable sugars, such as hexoses (glucose) and pentoses (xylose and arabinose)—the major sugars present in these raw materials (Saucedo-Luna et al. 2011, Moremi et al. 2020). In the hydrolysis step, the hydrolases used are expensive, which increases the final product cost. Furthermore, current industrial yeasts may be unable to metabolize pentoses. To overcome these bottlenecks, one strategy involves screening yeasts capable of efficiently utilizing the products of lignocellulosic biomass hydrolysis, especially xylose and arabinose, or even capable of hydrolyzing the polysaccharides cellulose and hemicellulose (Sukpipat et al. 2017, Moremi et al. 2020, Pooja et al. 2021, Albarello et al. 2023, Alencar et al. 2023).

Since several strains of *M. caribbica* have been isolated from ecological niches involving plant biomass (abundant in fermentable sugars), strains that can utilize the sugars present there have been explored, especially with a view to the consumption and fermentation of pentoses (Hawaz et al. 2022, Alencar et al. 2023). Giehl et al. (2025), for example, demonstrated that four *M. caribbica* isolates—namely CHAP-277, CHAP-280, CHAP-281, and CHAP-282—were able to grow on both arabinose and xylose as carbon sources. In another study, the strain of *M. caribbica* 5XY2, when cultivated in media containing xylose or arabinose as carbon sources, fermented both sugars and produced ethanol; however, the main products of these metabolisms were xylitol from xylose and arabitol from arabinose (Sukpipat et al. 2017).

The *M. caribbica* ability to use different lignocellulosic substrates has also been described. In this sense, Pooja et al. (2021) evaluated the fermentative potential of the strain *M. caribbica* M72 in rice straw hydrolysate: the strain produced 24.36 g/l of ethanol, with an efficiency of 95% and a productivity of 0.50 g/l/h. The authors emphasize that these values ​​were possibly achieved thanks to the utilization of the sugars present in the hydrolysate (including hexoses and pentoses) by the yeast cells (Pooja et al. 2021). At this point, regarding the co-fermentation of different sugars, Alencar et al. (2023) suggest integrating biomass hydrolysates into the conventional E1G matrix as a strategy. They argue that an ethanol industry focused exclusively on alternative substrates is not economically viable. Therefore, the authors tested the fermentative capacity of *M. caribbica* URM8365 in media with different proportions of sugarcane molasses and hydrolysates (sugarcane bagasse, sweet sorghum bagasse, and cactus pear biomass). The results of the mixed technique showed that ethanol production from xylose was only achieved due to the presence of glucose in the medium (Alencar et al. 2023). In fact, there seems to be no consensus regarding the fermentation of xylose to ethanol by *M. caribbica*, since there are reports of the absence of ethanol during fermentation and accumulation of xylitol (Trichez et al. 2019, Tadioto et al. 2022, Alencar et al. 2023). On the other hand, the diversification of metabolites generated from the fermentation of lignocellulosic substrates is extremely attractive from a multi-product biorefinery perspective, especially when the final product is high-value-added, such as xylitol.

In industry, xylitol is produced by chemical hydrogenation of xylose, a costly process that requires extreme temperatures and pressures, as well as the use of toxic catalysts. This molecule has applications in food, pharmaceuticals, and dentistry, and therefore has significant biotechnological value (Alkim et al. 2016, Saputra et al. 2020, Kaur et al. 2023, Singh et al. 2024). Alternatively, xylitol can be produced by microbiological processes, which are particularly interesting in the context of the bioeconomy, as yeasts can convert low-cost substrates into value-added compounds (Trichez et al. 2019, Kaur et al. 2023). Similarly, arabitol is a polyol structurally related to xylitol and can also be produced by biotechnological routes. This compound has attracted attention due to its applications in the food and pharmaceutical industries, where it serves as a low-calorie sweetener (Kordowska-Wiater 2015).

Regarding microbial xylitol production, species such as *M. guilliermondii, Candida tropicalis*, and *Spathaspora amazonensis* have already been characterized as excellent producers of this metabolite (Trichez et al. 2019). In addition to these species, *M. carribica* also stands out as a promising micro-organism, since research suggests that this biomolecule is the main product of this yeast metabolism when using xylose as a carbon source (Sukpipat et al. 2017, Moremi et al. 2020, Kaur et al. 2023). In this sense, in fermentation assays under limited oxygen conditions, the strain *M. caribbica* JA9 exhibited a higher yield (0.54 ± 0.11 g_xylitol_/g_xylose_) than the yeast *M. guilliermondii* (0.44 ± 0.02 g_xylitol_/g_xylose_), considered an excellent producer of this polyol (Trichez et al. 2019). A good yield was also reported by Sukpipat et al. (2017) (0.60 g_xylitol_/g_xylose_), after bioconversion of 80% of the xylose in the medium, under oxygen restriction.

Under optimized xylitol production conditions in a bioreactor fermentation, *M. caribbica* CP02 strain demonstrated the ability to ferment a rice straw hydrolysate with high xylose concentrations, yielding 0.63 g_xylitol_/g_xylose_, even in the presence of inhibitors. This suggests the resistance and robustness of this fermentation. Furthermore, aeration conditions proved to be a determining parameter, as a high oxygen supply results in greater cell biomass, while a microaerobic environment favors xylitol production (Singh et al. 2024). However, Kaur et al. (2023) highlight that, although this process is tolerant to acetic acid at pH 5.5 and 6.5, there is a decline in xylitol titer depending on the increasing concentration of the inhibitor. Therefore, approaches such as evolutionary adaptation in non-detoxified rice straw biomass hydrolysate have been proposed, which increase natural tolerance to inhibitors. Using this strategy, the authors documented an 8.33% increase in xylitol production compared to the non-developed parental strain.

Genomic sequencing of *M. caribbica* has allowed the identification of genes associated with xylose metabolism, some of which are exclusive to xylolytic yeasts and absent in *S. cerevisiae* (Trichez et al. 2019). Following the transport of the pentose (mediated by specific transporters), in the cytoplasmic environment, xylose is initially reduced to xylitol by the enzyme xylose reductase (XR), usually dependent on NADPH. In the next step, xylitol is oxidized to xylulose by xylose dehydrogenase (XDH), which may have a higher affinity for NAD^+^ than for NADP^+^. Finally, xylulose is phosphorylated by xylulokinase (XK), and the resulting xylulose-5P is directed into the pentose phosphate pathway (PPP). Thus, the first two reactions exhibit preferences for distinct cofactors, creating a redox imbalance. This cofactor mismatch is significant because it drives many yeasts to accumulate xylitol—the primary reaction product (Sukpipat et al. 2017, Patiño et al. 2019, Trichez et al. 2019, Alencar et al. 2023). Based on the aspects mentioned, Trichez et al. (2019) evaluated the activity and cofactor preferences of the XR and XDH enzymes in different yeasts, including *M. caribbica*. In the study, XR activity was NADPH-dependent, whereas XDH activity was NAD^+^-dependent, which is consistent with several studies that associate this species with high xylitol productivity.

The potential use of *M. caribbica* in biorefineries is not limited to pentose fermentation. Strains of this yeast have demonstrated tolerance to fermentation inhibitors, such as furans, weak acids, and phenolic compounds (Moremi et al. 2020, Tadioto et al. 2022). These molecules are released especially during the biomass pretreatment stage and are associated with low product yield, as they hinder yeast metabolism (Perna et al. 2018, Alencar et al. 2023, Kaur et al. 2023). In this context, different *M. caribbica* strains (namely strains D28L3, D14W2, D28L4, D14YE6, Mu 2.2f, D14YE1, D14YE2, and D4WPO1) maintained their growth capacity in media with arabinose supplemented with 3 g/l of acetic acid. Among them, D28L3, D14YE1, D14YE6, and Mu 2.2f, after evolutionary adaptation experiments, showed significant improvements in ethanol and arabitol production, also in the presence of the inhibitor acetic acid (Moremi et al. 2020). In another study, the concomitant effect of the main inhibitors present in hydrolysates—acetic acid, formic acid, furfural, and hydroxymethylfurfural—was tested using the strain *M. caribbica* CHAP-204. Even under these hostile conditions, in assays with optimized sugar concentration (∼55–85 g/l) and pH (∼6.0–8.5), the metabolism of this strain resulted in the production of ethanol and xylitol (Albarello et al. 2023). Indeed, at near-neutral pH, this yeast assimilates greater amounts of xylose, which may be correlated with an attenuation of the toxic effects of inhibitors, such as acetic acid (Kaur et al. 2023). Additionally, studies indicate that certain strains can grow at 37°C–40°C and produce lignocellulolytic enzymes, including amylase, xylanase, and cellulase. These characteristics, in turn, are particularly interesting, since they can be exploited in simultaneous saccharification and fermentation processes (Moremi et al. 2020, Rattanapatpokin et al. 2020, Ali et al. 2021, Albarello et al. 2023).

It is also important to highlight that the ability to metabolize arabinose and produce ethanol and/or arabitol, as demonstrated in the works of Moremi et al. (2020) and Sukpipat et al. (2017), is another important characteristic of *M. caribbica*. The yeast *S. cerevisiae*, for example, is not naturally capable of using arabinose, requiring extensive genetic modifications to allow its assimilation (Wang et al. 2017). Even among non-conventional yeasts, only a few genera can metabolize arabinose. The ability to ferment this sugar into ethanol is even less common than the ability to grow in the presence of this pentose (Ruchala and Sibirny 2021). Therefore, the ability of *M. caribbica* to convert arabinose into bioproducts represents an advantage in the context of biorefineries aiming at the integral utilization of pentoses from lignocellulosic biomass.

Table 1 summarizes key findings from fermentation studies employing *M. caribbica* strains. Across diverse experimental conditions, specific strains demonstrate the capacity to produce valuable metabolites despite exposure to inhibitory compounds and stress factors including elevated temperatures, high substrate concentrations, and acidic pH environments. Furthermore, *M. caribbica* strains are also being evaluated for their ability to modulate the volatile compound profile of fermented beverages, as detailed in the following section.

**Table 1:** Biotechnological products and fermentative performance of *M. caribbica* reported in the literature.

Bioproduct	*M. caribbica* strains analyzed in the referenced studies	Feedstock or media	Key fermentative parameters	Main remarks	Reference
Acetaldehyde	CCMA 0198 (UFLA CAF733)	Vinasse and molasses	n.a.	The *M. caribbica* strain CCMA 0198 was identified as the best acetaldehyde producer among all yeasts tested. However, this compound was determined solely based on its retention time in gas chromatography.	Coimbra et al. (2021)
		Sugarcane juice	Maximum titer in pure culture: 0.33 mg/l	Acetaldehyde production by *M. caribbica* was higher in pure culture than in co-culture with *S. cerevisiae*.	Duarte, Amorim and Schwan (2013)
		Sugarcane juice	Maximum titer in co-culture with *S. cerevisiae*: 38.73 mg/l	The use of a mixed inoculum (*M. caribbica* + *S. cerevisiae*) resulted in a higher average acetaldehyde concentration (38.73 mg/l) compared with fermentation carried out solely with *S. cerevisiae* (27.06 mg/l)	Amorim et al. (2016)
Arabitol	5XY2	Rich medium supplemented with glucose, arabinose, or xylose	Maximum titer: 30.3 g/l Maximum yield: 0.61 g_arabitol_/g_arabinose_	This was the first demonstration of arabitol production by *M. caribbica*. It suggests that this yeast has the enzymes involved in the arabinose catabolic pathway, such as arabitol dehydrogenase.	Sukpipat et al. (2017)
	D28L3, D14W2, D28L4, D14YE6, Mu2.2f, D14YE1, D14YE2, D4WPO1	Rich medium supplemented with arabinose + 3 g/l of acetic acid	Maximum titer: 26.7 g/l Maximum yield: 0.9 g_arabitol_/g_arabinose_	The adapted strain *M. caribbica* Mu2.2f exhibited a yield close to the theoretical maximum for arabitol, even in the presence of fermentation inhibitors such as acetic acid, highlighting it as an excellent alternative for the production of this polyol.	Moremi et al. (2020)
Ethanol	5XY2	Rich medium supplemented with glucose, arabinose or xylose	Maximum titer: 21.7 g/l Maximum yield: 0.45 g_ethanol_/g_glucose_	The ethanol yield observed corresponds to 88% of the maximum theoretical fermentation yield, demonstrating the yeast’s ability to efficiently convert glucose into ethanol.	Sukpipat et al. (2017)
	CCMA 0198 (UFLA CAF733)	Sugarcane juice	Maximum titer: 37.8 g/l Maximum yield: 0.37 g_ethanol_/g_sugar_	The use of *M. caribbica* in co-culture with *S. cerevisiae* resulted in higher ethanol concentration among all mixed inocula evaluated in the experiment, reaching 75.37 g/l.	Duarte, Amorim and Schwan (2013)
		Sugarcane juice and pineapple pulp (70:30)	Maximum titer: 83.8 g/l Maximum yield: 0.49 g_ethanol_/g_sugar_	*Meyerozyma caribbica* achieved a high ethanol yield (96.10 of the theoretical maximum), indicating an efficient conversion of fermentable sugars into ethanol.	Ribeiro et al. (2015)
		Vinasse and molasses (70:30)	Maximum titer: 35.76 g/l Yield: 0.17 g_ethanol_/g_sugar_	The *M. caribbica* strain CCMA 0198 was identified as the highest ethanol producer among all yeast species tested. Yield was calculated from the data reported in the referenced study, considering the concentration of fermentable sugars at the beginning of fermentation.	Coimbra et al. (2021)
	CHAP-087, CHAP-091, CHAP-096, CHAP-103	Minimal medium supplemented with glucose, xylose, or corn hydrolysate	Maximum titer: 14.0 g/l Maximum yield: 0.51 g_ethanol_/g_glucose_	The maximum ethanol yield was achieved under microaerobic conditions, considering the glucose consumed from the hydrolysates.	Tadioto et al. (2022)
	CHAP-204	Rich medium supplemented with xylose and glucose + inhibitors (3.0 g/l acetic acid, 0.8 g/l formic acid, 0.2 g/l furfural and 0.1 g/l HMF)	Maximum titer: 17.6 g/l Maximum yield: 0.36 g_ethanol_/g_glucose_	Ethanol was produced even in the presence of several inhibitory compounds. Intermediate pH values ​​and sugar concentrations represent the best fermentation conditions.	Albarello et al. (2023)
	CHAP-242, CHAP-248	Media with variable concentrations of sucrose, peptone, and yeast extract, with pH adjusted to 3.0, 5.0, or 7.0.	Maximum titer: 54.3 g/l Maximum yield: 0.49 g_ethanol_/g_glucose_	The two strains showed high ethanol productivity and yield even at low pH and high sugar levels (adverse conditions).	Fenner et al. (2025)
	D28L3, D14W2, D28L4, D14YE6, Mu2.2f, D14YE1, D14YE2, D4WPO1	Rich medium supplemented with arabinose + 3 g/l of acetic acid	Maximum titer: 5.7 g/l Maximum yield: 0.34 g_ethanol_/g_arabinose_	The adapted strain *M. caribbica* Mu2.2f achieved satisfactory ethanol production in a bioreactor in the presence of 3 g/l acetic acid.	Moremi et al. (2020)
	JA9	Mineral medium supplemented with xylose or a mixture of xylose and glucose and hydrolyzed sugarcane biomass	Maximum titer: 5.7 g/l Maximum yield: 0.12 g_ethanol_/g_sugar_	Ethanol production by this strain was observed only when glucose was present in the culture medium. Xylose was directed toward xylitol accumulation.	Trichez et al. (2019)
	M72	Hydrolyzed rice straw	Maximum titer: 24.4 g/l Maximum yield: 0.48 g_ethanol_/g_sugar_	A high ethanol yield was obtained through fermentation in saccharified hydrolysate under optimized conditions. The yeast also showed tolerance to ethanol at a concentration of up to 8%.	Pooja et al. (2021)
	MJTm3	Sugarcane molasses	Maximum titer: 42.1 g/l % of theoretical yield: 89.42%	The strain stood out for its tolerance to various stress factors, as it maintained its growth capacity at high ethanol concentrations and temperatures up to 42°C. Also, during fermentation tests, it exhibited the highest ethanol production capacity.	Hawaz et al. (2022)
	URM 8365	Sugarcane molasses + hydrolysates (sugarcane bagasse, sweet sorghum bagasse and cactus pear biomass)	Maximum titer: 50.0 g/l Maximum yield: 0.49 g_ethanol_/g_sugar_	The URM 8365 strain demonstrated exceptional versatility, being able to produce ethanol with high efficiency from both first-generation (1 G) and second-generation (2 G) feedstocks.	Alencar et al. (2023)
2-phenylethanol (2-PE)	CCMA 0198 (UFLA CAF733)	Sugarcane juice	Maximum titer in pure culture: 10.72 mg/l	The production of 2-phenylethanol by *M. caribbica* was enhanced when it was cultivated in association with *S. cerevisiae*. Its use in co-culture increased this value to 24.49 mg/l.	Duarte, Amorim and Schwan (2013)
		Sugarcane juice and pineapple pulp (80:20)	Maximum titer in pure culture: 120.67 mg/l	The use of *M. caribbica* resulted in a notable increase in the concentrations of this compound compared with cultures involving *S. cerevisiae*.	Ribeiro et al. (2015)
		Sugarcane juice	Maximum titer in co-culture with *S. cerevisiae*: 15.89 mg/l	The mixed inoculum (*M. caribbica* + *S. cerevisiae*) showed higher efficiency for 2-phenylethanol production than the pure *S. cerevisiae* culture.	Amorim et al. (2016)
	CHAP-242, CHAP-248	Media with variable concentrations of sucrose, peptone, and yeast extract, with pH adjusted to 3.0, 5.0, or 7.0.	n.a.	2-phenylethanol production was favored by low nitrogen availability. However, this compound was determined semi-quantitatively, based on its relative peak area, in gas chromatography.	Fenner et al. (2025)
	WUT28	Whey, glucose, and L-phenylalanine	Maximum titer: 1.65 g/l	*Meyerozyma caribbica* stood out for its rare metabolic ability among yeasts to utilize lactose as a carbon source, which makes it an ideal candidate for the valorization of industrial residues such as whey. When cultivated in a whey-based medium supplemented with glucose and phenylalanine, the strain achieved a 2-PE production of 1.33 g/l.	Chreptowicz et al. (2018)
Xylitol	5XY2	Rich medium supplemented with glucose, arabinose or xylose	Maximum titer: 20.2 g/l Maximum yield: 0.60 g_xylitol_/g_xylose_	The xylitol yield achieved indicates the yeast’s efficient conversion of xylose into xylitol under semi-anaerobic conditions.	Sukpipat et al. (2017)
	CHAP-087, CHAP-091, CHAP-096, CHAP-103	Minimal medium supplemented with glucose, xylose, or corn hydrolysate	Maximum titer: 6.4 g/l Maximum yield: 0.32 g_xylitol_/g_xylose_	An oxygen-limited environment prevented the yeast from metabolizing xylose. In aerobic conditions, this carbon source was consumed and converted into xylitol, without ethanol production. In hydrolysate fermentation, the pentose was not completely consumed and there was no accumulation of xylitol (probably due to the presence of acetic acid).	Tadioto et al. (2022)
	CHAP-204	Rich medium supplemented with xylose and glucose + inhibitors (3.0 g/l acetic acid, 0.8 g/l formic acid, 0.2 g/l furfural and 0.1 g/l HMF)	Maximum titer: 13.1 g/l Maximum yield: 0.35 g_xylitol_/g_xylose_	Xylitol was produced even in the presence of several inhibitory compounds. Intermediate pH values ​​and sugar concentrations represent the best fermentation conditions.	Albarello et al. (2023)
	CP02	Hydrolyzed rice straw	Maximum titer: 67.0 g/l Maximum yield: 0.77 g_xylitol_/g_xylose_	During fermentation in a batch bioreactor, with optimized parameters (temperature, pH, agitation, and initial inoculum), this isolate produced xylitol in high yields, even under adverse conditions such as the presence of inhibitors and high sugar levels.	Singh et al. (2024)
	JA9	Mineral medium supplemented with xylose or a mixture of xylose and glucose and hydrolyzed sugarcane biomass	Maximum titer: 17.3 g/l Maximum yield: 0.54 g_xylitol_/g_xylose_	Xylitol was the main product of this yeast’s metabolism, even during fermentation with non-detoxified hydrolysate.	Trichez et al. (2019)
	MZ05761	Hydrolyzed rice straw	Maximum titer: 34.9 g/l Maximum yield: 0.69 g_xylitol_/g_xylose_	Xylitol production increased by 8.33% after evolutionary adaptation of this strain in non-detoxified rice straw biomass hydrolysate.	Kaur et al. (2023)
	URM 8365	Sugarcane molasses + hydrolysates (sugarcane bagasse, sweet sorghum bagasse and cactus pear biomass)	Maximum titer: 11.7 g/l Maximum yield: 0.30 g_xylitol_/g_xylose_	A portion of the xylose was consumed and converted into ethanol only when glucose was present. Xylitol production was impaired at acetic acid concentrations of 2.5 g/l in the hydrolysates, a problem overcome after removing this inhibitor.	Alencar et al. (2023)
Other volatile organic compounds (VOCs)	CCMA 0198 (UFLA CAF733)	Sugarcane juice	Maximum production of: 2-methyl-1-butanol + 3-methyl-1-butanol: 121.53 mg/l Propanol: 0.24 mg/l Linalool: 0.12 mg/l Decanoic acid: 2.67 mg/l Octanal: 0.28 mg/l	In co-culture, the mixture of *M. caribbica* and *S. cerevisiae* produced the highest total concentration of desirable compounds among all groups tested, including ethyl esters (0.29 mg/l), acetates (0.72 mg/l), and monoterpene alcohols (0.20 mg/l).	Duarte, Amorim and Schwan (2013)
		Sugarcane juice and pineapple pulp	Maximum production of: 3-methyl-1-butanol: 150.36 mg/l 2-methyl-1-propanol: 32.45 mg/l 1-propanol: 18.33 mg/l Verbenone: 4.46 mg/l Phenyl acetate: 35.89 mg/l Ethyl octanoate: 7.76 mg/l Phenylethyl acetate: 5.40 mg/l Ethyl acetate: 3.49 mg/l Propyl butyrate: 2.28 mg/l Isobutyric acid: 14.10 mg/l Octanoic acid: 11.34 mg/l Decanoic acid: 6.27 mg/l Nonanoic acid: 4.94 mg/l β-Citronellol: 1.44 mg/l	The VOCs produced by *M. caribbica* are essential for enhancing the sensory quality of the Beverage produced, surpassing in several aspects the results obtained with pure cultures of *S. cerevisiae*.	Ribeiro et al. (2015)
		Sugarcane juice	Maximum production of: Ethyl acetate: 53.72 mg/l 2-methyl-1-propanol: 319.80 mg/l 2-methyl-1-butanol: 130.53 mg/l 3-methyl-1-butanol: 743.84 mg/l Furfuryl alcohol: 1.07 mg/l	The cachaça produced by the mixed culture showed the highest concentration of volatile compounds.	Amorim et al. (2016)

n.a., not available in the referenced study.

## Other value-added bioproducts

In addition to producing ethanol, xylitol, or arabitol, *M. caribbica* strains have also demonstrated the ability to synthesize a variety of other compounds, some of which can even add value to the final products of traditional fermentation processes (see Table 1). In the production of fermented beverages, for example, *M. caribbica* has been tested in co-cultures with *S. cerevisiae*, forming consortia that result in more complex aromatic profiles and add value to the beverages. In the production of a fermented beverage from sugarcane and pineapple, the combination of *S. cerevisiae* and *M. caribbica* increased the concentrations of desirable volatile compounds in the final product, such as 2-phenylethanol, 2-methyl-1-propanol, 3-methyl-1-butanol, ethyl acetate, and phenylethyl acetate (Ribeiro et al. 2015). In a similar study, Amorim et al. (2016) demonstrated that, in the production of cachaça (sugar cane spirit), a mixed inoculum of *S. cerevisiae* and *M. caribbica* resulted in a beverage with a higher concentration of volatile compounds associated with good sensory descriptors, including 2-phenylethanol, compared to the beverage obtained from fermentation using only *S. cerevisiae*. In another study, cachaça produced with mixed *M. caribbica*/*S. cerevisiae* cultures showed increased concentrations of desirable volatile compounds, including ethyl hexanoate, 2-phenylethanol, linalool, nonanoic acid, ethyl butyrate, and phenylethyl acetate, indicating that this mixed inoculum could produce a beverage with a favorable flavor profile (Duarte, Amorim and Schwan 2013). In the three studies mentioned above, the *M. caribbica* strain used was CCMA0198 (original name UFLA CAF733), isolated from coffee fermentation (Duarte, Amorim and Schwan 2013).

Beyond its applications in fermented beverages, *M. caribbica* strains have been explored for the direct biosynthesis of volatile compounds for other purposes. In this context, 2-phenylethanol stands out. The strain *M. caribbica* WUT28, isolated from dark grapes, was able to produce 1.33 g/l of 2-phenylethanol from a culture medium containing 3% whey, 2% glucose, and 0.5% L-phenylalanine (Chreptowicz et al. 2018). Furthermore, Fenner et al. (2025) demonstrated that two strains of *M. caribbica* isolated from *Scaptotrigona postica* bees had a strong capacity to produce 2-phenylethanol, with production higher at high sugar concentrations and low nitrogen availability, which can be readily achieved using low-cost residual raw materials.

*Meyerozyma caribbica* also exhibits the ability to generate other metabolites of chemical interest, such as acetaldehyde. In this sense, Coimbra et al. (2021) used vinasse from the production of distilled beverages, along with molasses, as a fermentative medium for *M. caribbica*, and the yeast produced mainly acetaldehyde. This volatile metabolite is widely used as a fine chemical in the food and flavor industries, and can also serve as a platform for the synthesis of acetic acid, pyridines, and 1,3-butylene glycol (Mengers et al. 2022).

Lipid accumulation, especially for biodiesel synthesis, is also being tested using strains of *M. caribbica* due to their ability to accumulate lipids. Chebbi et al. (2019) demonstrated that a strain of *M. caribbica*, when cultivated in a fed-batch bioreactor with glycerol as a carbon source, produced biomass with a microbial oil content of 48.14% (w/w). Similarly, Ali et al. (2021) isolated a strain of *M. caribbica* from the gut of wood-eating termites that exhibited a lipid accumulation capacity of up to 47,25% (w/w). In another study, Kumar et al. (2020) optimized a culture medium and achieved a lipid yield of 0.49 g/g with a strain of *M. caribbica* isolated from an oil mill area; these authors also found that the lipid produced by the cells of this yeast has a good ratio (44.5 : 55.9) of saturated to unsaturated fatty acids, which is favorable for biodiesel applications.

Interestingly, although this topic has been little to almost not explored for *M. caribbica*, cell size plays a crucial role in lipid accumulation (Blank et al. 2020, Li et al. 2026) and cell disruption (Solecki et al. 2021, Zainuddin et al. 2021) for lipid recovery. Larger or growth-arrested cells tend to accumulate more lipids, possibly because lower division rates result in reduced dilution of lipid storage, while fluctuations in cell size linked to the cell cycle can influence the timing and extent of lipid droplet formation (Morimoto et al. 2022). Addressing this aspect should be a key focus of future studies aimed at optimizing fermentation conditions to maximize lipid yields by *M. caribbica*. Moreover, the efficiency of cell disruption techniques such as bead milling, high-pressure homogenization, ultrasonication, pyrolysis, or acid hydrolysis is affected by the inherent cell size distribution and cell wall structure (Gorte et al. 2020, Kot et al. 2020, Solecki et al. 2021, Zainuddin et al. 2021, Planonth and Chantarasiri 2022, Bhowmick et al. 2024). Tailoring the disruption protocol to the specific characteristics of *M. caribbica* can enhance lipid recovery while balancing energy consumption and process scalability.

## Biocontrol applications of *M. caribbica* in plant disease management

*Meyerozyma caribbica* has emerged as a potent biocontrol yeast for managing postharvest diseases of fruits and vegetables. It has demonstrated broad efficacy against a range of fungal pathogens. For example, strains of *M. caribbica* effectively suppress *Colletotrichum* spp. (the causative agents of anthracnose) in tropical fruits (Navarro-Herrera and Ortíz-Moreno 2020, Moreno-Hernandez et al. 2024), inhibit *Penicillium expansum* (which causes blue mold) and even reduce patulin mycotoxin contamination in apples and other fruits (Dudaš et al. 2026), and control *Alternaria alternata* (the causative agent of black spot rot) in jujube fruits (Deng et al. 2022). In these hosts, applying *M. caribbica* significantly lowers disease incidence and lesion development compared to untreated controls (Deng et al. 2022, Dudaš et al. 2026). It can be applied either preharvest (e.g. as a field treatment) or postharvest (e.g. as a wound treatment or coating) and has shown protective effects across diverse crops when properly formulated (Dudaš et al. 2026).

On papaya anthracnose lesions, field-oriented trials with encapsulated formulations with *M. caribbica* showed inhibitory activity against anthracnose (*Colletotrichum gloeosporioides*) *in vitro* and *in vivo* (Aguirre-Güitrón et al. 2022). In temperate fruits, *M. caribbica* suppressed *Botrytis cinerea* (grey mold) and *Rhizopus* rots on strawberries. It significantly reduced grey mold incidence and lesion size, partly by inducing host defense enzymes, such as β-1,3-glucanase, in the fruit (Zhao et al. 2012). On peaches, *M. caribbica* treatments similarly reduced soft rot caused by *Rhizopus stolonifer*, correlating with enhanced peroxidase, catalase and phenylalanine ammonia-lyase activity in the fruit tissue (Xu et al. 2013). In kiwifruit, a high dose (10^9^ cells/ml) of *M. caribbica* completely prevented blue mold decay, whereas a lower dose yielded a significant reduction in the disease prevention (Qiu et al. 2022). Notably, *M. caribbica* can thrive as a phyllosphere yeast, enabling preharvest use. In this context, a stress-tolerant strain applied in the field provided significant protection against anthracnose (84.9% reduction in banana lesions and up to 91.8% reduction in strawberry disease incidence) (Baral et al. 2025).

Table 2 summarizes major examples of *M. caribbica* used as a biocontrol agent on various crops against diverse pathogens. Key formulations and outcomes are noted, along with representative references from recent studies.

**Table 2:** Applications of *M. caribbica* as biocontrol of plant pathogens.

Crop	Target pathogen	Formulation/treatment	Biocontrol outcome	Reference
Mango	*Colletotrichum gloeosporioides* (fungus; anthracnose)	Fresh yeast cell suspension (spray)	86.7% reduction in anthracnose lesion	(Bautista-Rosales et al. 2013)
Mango	*C. gloeosporioides*	Spray-dried formulation (protective agent trehalose at 7.75%)	53.4% reduction in anthracnose incidence	(Aguirre-Güitrón et al. 2019)
Papaya	*C. gloeosporioides*	Encapsulated *M. caribbica* (electrosprayed powder)	Anthracnose effectively controlled. Encapsulated yeast inhibited lesions similar to fresh cells	(Aguirre-Güitrón et al. 2022)
Avocado	*C. gloeosporioides*	Edible sodium alginate coating	Significant reduction of anthracnose	(Iñiguez-Moreno et al. 2020)
Kiwifruit	*Penicillium expansum* (fungus; blue mold rot)	Yeast cell suspension	100% reduction of blue mold incidence at 10^9^ CFU/ml	(Qiu et al. 2022)
Jujube (Chinese red date)	*Alternaria alternata* (fungus; black spot rot)	Yeast cell suspension (with biofilm promoter: 1 mM phenylalanine)	Significant suppression of black spot rot. *M. caribbica* treatment reduced decay, formed biofilms in wounds, and adhered to pathogen hyphae	(Deng et al. 2022)
Banana	*Colletotrichum musae* (fungus; anthracnose)	Yeast in mannitol-based formulation (osmoprotectant added)	84.9% reduction in anthracnose lesion size on banana fruit. Formulation improved yeast stress tolerance and efficacy under tropical conditions	(Baral et al. 2025)
Strawberry	Mixed natural infection	Yeast in mannitol-based formulation (osmoprotectant added)	91.8% decrease in overall postharvest decay incidence on strawberries	(Baral et al. 2025)
Peach	*Rhizopus stolonifer* (fungus; soft rot)	Yeast cell suspension (antagonist coating)	Marked reduction in *Rhizopus* rot incidence and lesion diameter	(Xu et al. 2013)

Antagonistic *M. caribbica* yeasts employ multiple modes of action at the molecular level to suppress postharvest pathogens. A key mechanism is rapid surface colonization and biofilm formation, since *M. caribbica* can quickly establish itself in fruit wounds and adhere tightly to pathogen hyphae, secreting extracellular polymers that form a biofilm matrix (Bautista-Rosales et al. 2013). Quorum-sensing signals appear to regulate these processes, since *M. caribbica* naturally secretes the volatile 2-phenylethanol, an aromatic alcohol derived from phenylalanine metabolism, which acts as a quorum-sensing molecule to enhance yeast adhesion and biofilm development (Wu et al. 2024). Experimental supplementation with exogenous L-phenylalanine, a precursor of 2-phenylethanol, dramatically increases 2-phenylethanol production by *M. caribbica*, correlating with thicker biofilm formation and improved biocontrol efficacy against fruit pathogens (Deng et al. 2022). Also, Zhang et al. (2023) provided evidence that the production of 2-phenylethanol by *M. caribbica* may play a role in its antagonism of phytopathogenic fungi. The authors isolated a new 2-phenylethanol-producing *M. caribbica* strain (NJC41) that effectively suppressed the soybean pathogen *Corynespora cassiicola*. The antagonistic activity of this yeast was attributed to the secretion of antifungal 2-phenylethanol, with 44.6 μg/ml detected in its culture filtrates, and to competition for space and nutrients with the pathogen (Zhang et al. 2023). The yeast also has the ability to produce inhibitory volatile organic compounds (VOCs) such as alcohols or esters, often inhibiting pathogen spore germination and growth at a distance (Herrera-Balandrano et al. 2023). Notably, genomic analyses suggest that *M. caribbica* is well-equipped for such metabolism, possessing genes for robust respiration and alcohol oxidation. For example, copy number gains in cytochrome-B and alcohol oxidase genes may enable enhanced production of reactive aldehydes and hydrogen peroxide, creating a hostile oxidative environment for competing fungi (Vicente et al. 2025)

In addition to VOCs production, *M. caribbica* expresses hydrolytic enzymes and other antifungal factors that contribute to its biocontrol activity. Transcriptomic and proteomic studies of closely related yeast species indicate upregulation of genes encoding cell wall-degrading enzymes, such as chitinases, exoglucanases, and endoglucanases, during antagonistic interactions (Herrera-Balandrano et al. 2023). Secretion of these lytic enzymes enables *M. caribbica* to directly attack fungal pathogens. The yeast can parasitize hyphae by enzymatically degrading the pathogen’s cell wall, as evidenced by microscopic observations of yeast cells attached to and lysing (Deng et al. 2022, Herrera-Balandrano et al. 2023). *Meyerozyma caribbica* also likely upregulates high-affinity nutrient transporter genes under nutrient-limited wound environments, outcompeting pathogens for essential elements. For example, competition for iron, often mediated by secreted siderophores or membrane transporters, is a known mechanism in closely related yeast species that deprives fungi of this growth-limiting micronutrient (Herrera-Balandrano et al. 2023). Similarly, duplication and enrichment of genes for nitrogen metabolism, such as urea hydrolysis and amino acid permeases, in *M. guilliermondii* genomes suggest an adaptive strategy to dominate nutrient-limited niches (Vicente et al. 2025).

Equally important is the indirect mechanism by which *M. caribbica* induces resistance in the host fruit, and recent molecular evidence has illuminated this aspect. When *M. caribbica* colonizes a fruit, it can act as an elicitor of the plant’s innate defense pathways, essentially priming the host tissue to better resist pathogens. Treated fruits consistently show enhanced activity of defense-related enzymes and upregulation of defense genes. For example, Zhang et al. (2022) demonstrated that *M. caribbica* triggers a suite of defense responses in cherry tomatoes against *Alternaria* rot. Enzymatic assays showed higher activity of pathogenesis-related (PR) proteins, such as chitinases and β-1,3-glucanases, in *M. caribbica*-treated tomatoes, as well as elevated levels of polyphenol oxidase and peroxidase, enzymes involved in strengthening host tissues (Zhang et al. 2022). Simultaneously, genes in the phenylpropanoid secondary metabolism pathway were strongly induced. Transcripts for phenylalanine ammonia-lyase (PAL) and cinnamate-4-hydroxylase, key enzymes for synthesizing phenolic compounds and lignin, were upregulated, leading to increased accumulation of total phenolics, flavonoids, and lignin in the fruit. These compounds strengthen cell walls and create antimicrobial conditions, thereby curtailing pathogen growth.

In addition to direct antagonistic mechanisms, *M. caribbica* has been shown to induce systemic resistance in host plants. Zhao et al. (2023) demonstrated that *M. caribbica* treatment in postharvest kiwifruit activated a wide range of defense-related pathways, including flavonoid biosynthesis, phenylpropanoid metabolism, and the plant-pathogen interaction pathway. Transcriptomic analysis revealed that genes involved in signal transduction, antioxidant activity, cell wall reinforcement, and secondary metabolite production were significantly upregulated. These findings highlight the ability of *M. caribbica* to enhance host immunity through molecular priming, providing an additional layer of biocontrol beyond pathogen inhibition (Zhao et al. 2023). Transcriptome analyses indicate that *M. caribbica* treatment can stimulate mitogen-activated protein kinase (MAPK) cascades, calcium-dependent signaling, and hormone-regulated defenses (e.g. jasmonate/ethylene pathways), which collectively orchestrate the expression of numerous PR genes (Zhang et al. 2022). As a result, *M. caribbica*-inoculated fruits exhibit higher expression of genes like CHI (chitinase), GLU (β-1,3-glucanase), and PR-1, and show an accumulation of defensive metabolites akin to a classic induced resistance response (Zhang et al. 2022, Yang et al. 2024).

**Figure 3:**
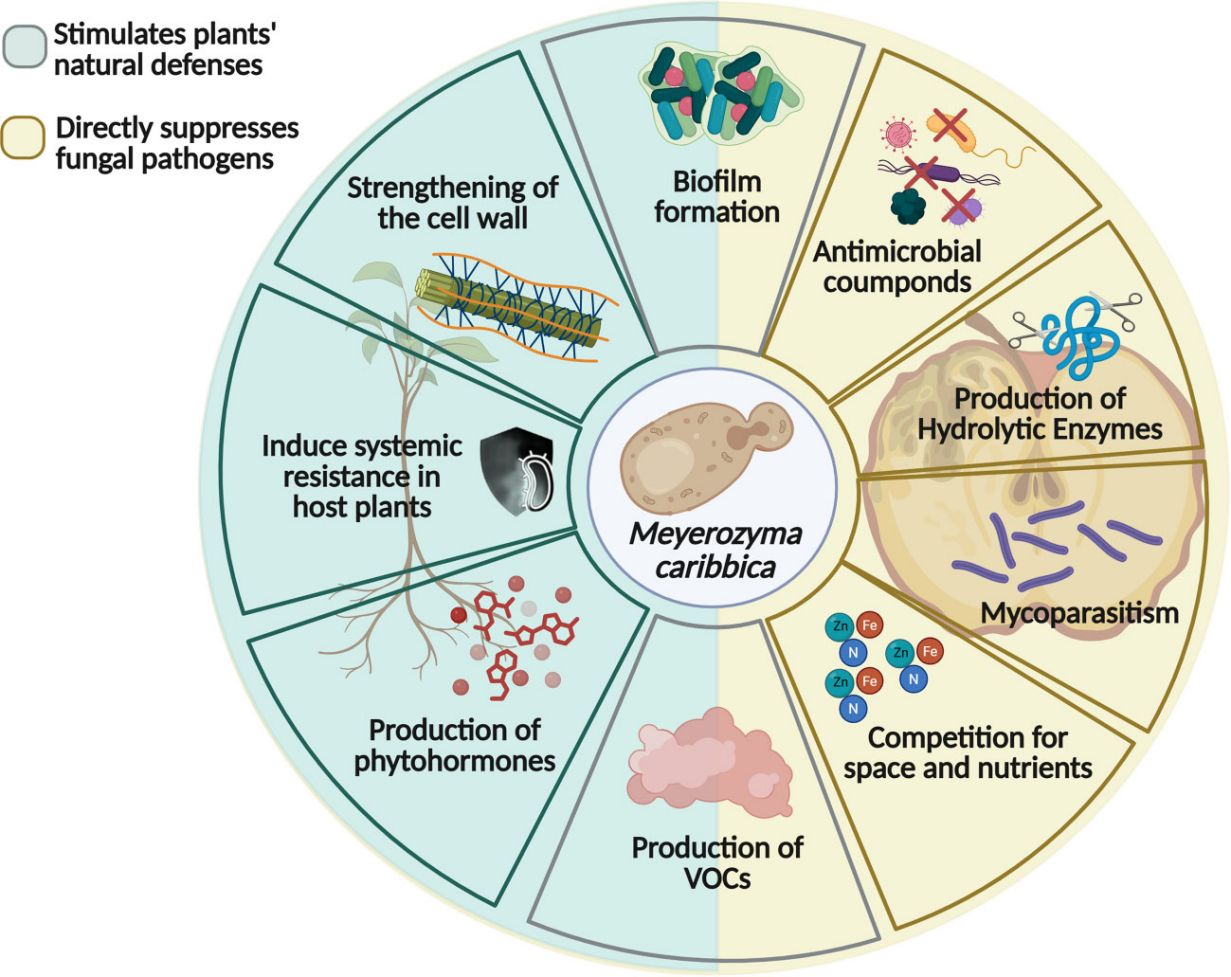
Representation of the main mechanisms through which *M. caribbica* interacts with phytopathogenic fungi and the plant host. The species combines direct antifungal actions, mediated by bioactive secretions and competitive interactions, with the ability to modulate plant defense responses, which underpins its performance as a biocontrol agent. VOCs: volatile organic compounds.

The main mechanisms underlying these biocontrol effects, including direct antifungal actions and the induction of host defense responses, are summarized in Fig. 3.

## Bioremediation and environmental applications

The intensification of industrial and agricultural activities has led to the buildup of persistent pollutants in soils and aquatic environments. These toxic substances hamper key ecological functions, reduce biodiversity, and limit agrarian productivity, making their removal one of today’s major environmental challenges (Rashid et al. 2023, Dhuldhaj et al. 2023, Gupta and Gandhi 2023). Several physiological and biochemical properties of *M. caribbica* support its use in bioremediation processes and environmental applications. Beyond its capacity to degrade complex organic compounds, pesticides, and heavy metals, the yeast can form synergistic consortia with other micro-organisms, establishing cooperative interactions that optimize pollutant degradation and resource utilization. In fact, its efficiency is strain dependent and reflects the integration of enzymatic and metabolic networks adapted to distinct environmental conditions (Amorim et al. 2018, Ali et al. 2021, Roy et al. 2024).

### Enzymatic degradation of organic pollutants

Among the extracellular enzymes involved in contaminant degradation, manganese peroxidase stands out, oxidizing lignin and other aromatic compounds. This enzyme converts metal ions into redox mediators that oxidize phenolic structures, promoting the cleavage of aromatic rings and the formation of less toxic products (Bilal et al. 2017, Ali et al. 2022). For example, this activity was observed in strain SSA1654, isolated from the gut of xylophagous termites, which exhibited high enzymatic activity and a strong ability to decolorize the azo dye Acid Orange 7 (AO7). In experiments conducted both in microbial consortia and in pure culture, the species demonstrated high efficiency in dye removal, confirming its ability to degrade complex aromatic compounds. According to the authors, spectroscopic and chromatographic analyses showed that the azo bonds were cleaved and the aromatic rings were broken down, resulting in the formation of less toxic products (Al-Tohamy et al. 2021, Ali et al. 2022)

The same enzymatic versatility also explains *M. caribbica*’s effectiveness in degrading highly persistent organochlorine pesticides, such as lindane (γ-HCH). Roy et al. (2024) reported that the NBRIHCH_Y2 strain, isolated from soil contaminated with hexachlorocyclohexane (HCH), was able to degrade this compound through enzymatic dechlorination and redox reactions, generating intermediates with lower levels of halogenation and toxicity. In combination with *Priestia megaterium*, the yeast exhibited enhanced degradation efficiency, suggesting metabolic synergy and complementarity between their enzymatic pathways.

*Meyerozyma caribbica* can act on organochlorine herbicides like 2,4-dichlorophenoxyacetic acid (2,4-D), whose chemical stability comes from chlorine atoms in the aromatic ring (Magnoli et al. 2023, Chen et al. 2024). The CHAP-248 isolate has shown partial removal of this herbicide, indicating the species' potential forinated compounds (Oliveira et al. 2025). Although the mechanism remains unclear, the transformation likely involves initial oxidative reactions followed by partial dechlorination, as described by Roy et al. (2024). These steps are common in fungi and yeasts that break down organochlorine herbicides, producing less toxic intermediates (Magnoli et al. 2023, Oliveira et al. 2025).

In addition to halogenated compounds, *M. caribbica* can also metabolize hydrocarbons, further reinforcing its potential to act on organic contaminants. This characteristic has been described in strains isolated under contrasting environmental conditions. Joshi-Navare et al. (2014) studied a strain obtained from industrial oily waste in India and demonstrated the production of a xylolipid biosurfactant, an amphiphilic compound capable of emulsifying hydrocarbons and increasing their bioavailability for degradation. Similarly, Martorell et al. (2017) isolated *M. caribbica* in Antarctic soils and detected psychrotolerant growth, the production of hydrolytic enzymes active at low temperatures (such as amylases, lipases, and proteases), and the assimilation of n-alkanes and diesel as carbon sources. This combination of enzymatic activity and ability to utilize hydrocarbons indicates that yeast can act effectively in bioremediation processes under adverse environmental conditions, including low temperatures and nutrient limitation (Martorell et al. 2019).

### Antioxidant modulation and plant stress tolerance

The physiological effects resulting from the action of *M. caribbica* in plant–micro-organism interactions indicate that its potential is not limited to chemical degradation but extends to the modulation of antioxidant responses and functional recovery of the rhizosphere. At this point, Roy et al. (2025) applied a *M. caribbica* + *P. megaterium* consortium to contaminated soil cultivated with *Ricinus communis* and observed, in addition to pesticide reduction, significant mitigation of oxidative stress and restoration of redox homeostasis in plants. Similar results were reported by Jan et al. (2019), who demonstrated that an endophytic isolate (SXSp1) increased corn plant tolerance to salt stress by activating antioxidant enzymes, accumulating phenolic compounds, and regulating phytohormones. These findings are consistent with the review by Herrera-Balandrano et al. (2023), which highlighted the ability of species of the genus *Meyerozyma* to induce resistance in host plants, thereby strengthening antioxidant metabolism and restoring cellular homeostasis even in the absence of pathogens.

### Biosorption and biofilm formation as adaptive strategies of *M. caribbica*

The metabolic versatility of *M. caribbica* also encompasses heavy metal immobilization, a passive process in which metal ions attach themselves to cell wall components. For example, Amorim et al. (2018) isolated a strain from mining water that was highly resistant to manganese and capable of completely removing Mn²⁺ ions through biosorption. Electron microscopy and spectroscopy analyses demonstrated metal deposition on the cell surface, a process compatible with ion adsorption to functional groups in the wall, such as carboxyl, hydroxyl, and phosphate groups. Furthermore, removal occurred independently of pH, suggesting high density and diversity of active binding sites. This process is consistent with the model proposed by Wang and Chen (2009), which suggests that yeasts can maintain high biosorption efficiency over a wide pH range due to the abundance of anionic groups involved in ion-exchange and complexation mechanisms.

Another factor contributing to the environmental efficiency of *M. caribbica* is the formation of biofilms, a characteristic observed in certain strains and considered an adaptive mechanism that favors cell attachment and confers greater tolerance to environmental stresses (Yin et al. 2019). In addition to its role in the biocontrol of phytopathogens, biofilm also contributes to the species' performance in different environmental systems. For example, Elsamahy et al. (2023) demonstrated that *M. caribbica* adhered to polymeric substrates, promoting the degradation of low-density polyethylene films. In mining effluents, Vázquez-González et al. (2024) found that yeast L6A2 formed only a thin biofilm in mining effluents when grown alone, but developed a thicker, more efficient structure with *P. megaterium*, removing up to 98% of Mn²⁺. These results demonstrate the versatility of biofilms, which improve the ecological stability of *M. caribbica* and support its use in environmental and biotechnological applications.

## Probiotic potential and food-related applications

Yeasts have long served in food manufacturing and are gaining renewed attention as probiotic candidates and multifunctional starter cultures. *Saccharomyces cerevisiae* var. *boulardii* is an established yeast probiotic, but recent surveys and experimental screens have uncovered promising non–*Saccharomyces* taxa from fermented products and spoiled foods (Fernández-Pacheco et al. 2021, Staniszewski et al. 2024). *Meyerozyma caribbica*, commonly isolated from fruits and fermentation environments, has emerged as a species of interest due to antifungal activity, metabolite production, and positive impacts when applied as an inoculum in beverages such as coffee (Herrera-Balandrano et al. 2023, Bernardes et al. 2024). Food wastes and by–products are attractive reservoirs for novel functional yeasts: they concentrate diverse microbes and are often rich in simple sugars and micronutrients that select for fermentative species.

### Probiotic assessment of *M. caribbica*

Food residues (fruit peels, pulp, and processing effluents) are ecological niches where facultative fermentative yeasts naturally thrive. Targeting these matrices increases the likelihood of recovering strains already adapted to complex substrates and stressors relevant to industrial use (Staniszewski et al. 2024). Moreover, sourcing strains from waste valorization pipelines aligns with circular economy goals and may yield candidates intrinsically tolerant to variable osmotic and oxidative conditions encountered during downstream processing and formulation. Importantly, isolates from food wastes have precedents of probiotic features and technological traits (Moonsamy et al. 2024). For example, *M. caribbica* isolated from pineapple peel exhibited high biomass formation and a specific growth rate comparable to the probiotic *S. cerevisiae* var. *boulardii*, supporting its potential as a functional yeast (Akter et al. 2021).

Studies investigating *Meyerozyma* spp. and other osmotolerant yeasts commonly report the use of fruit-processing residues and beverage effluents as productive sources of environmental isolates. These materials are typically handled under aseptic conditions and processed through serial dilution followed by cultivation on selective media, including YPD supplemented with antibiotics to suppress bacterial growth. Several works also highlight the use of acids or sugars to promote the recovery of osmotolerant species characteristic of the genus *Meyerozyma* (Akter et al. 2021, Shruthi et al. 2024). Species identification by ITS sequencing and confirmation with D1/D2 26S rRNA domain sequencing is recommended; multilocus approaches aid strain discrimination when precise typing is required for intellectual property or registration (D’Aquila et al. 2023). Rapid MALDI–TOF or matrix–assisted methods can complement sequencing in large screens. PCR–based strain-level techniques are practical for selecting candidates from diverse collections (Fernández-Pacheco et al. 2021).

Probiotic candidacy for yeasts requires a stepwise *in vitro* pipeline before *in vivo* or clinical work. Key assays include: (1) survival of gastrointestinal conditions; (2) adhesion and colonization proxies; (3) antimicrobial and antagonistic activities; (4) safety and absence of pathogenicity markers; (5) and functional assays. Assessments of gastrointestinal tolerance in yeasts typically involve exposing cells to simulated gastric conditions (pH 2–3 in the presence of pepsin) and subsequently to intestinal-like environments containing bile salts and pancreatin, with survival expressed as log reduction relative to untreated controls. Contemporary screening approaches often consider strains with limited viability loss—generally no more than a 1–2 log reduction under standardized conditions—as possessing robust resistance to gastrointestinal transit (Fernández-Pacheco et al. 2021). Consistent with these criteria, fruit-derived isolates of *M. caribbica* kept high viability after exposure to simulated gastric and intestinal stress, exhibiting survival patterns comparable to those of established probiotic yeasts (Amorim et al. 2018). Shruthi et al. (2024) demonstrated that the isolate MYSY22 exhibits acid and bile tolerance, high surface hydrophobicity, robust autoaggregation, antioxidant capacity, and cholesterol assimilation, all of which support survival within the gastrointestinal environment and the expression of beneficial effects. The strain also showed marked antagonistic activity against enteric pathogens, indicating potential to inhibit harmful bacteria under gastrointestinal-like conditions.

### *Meyerozyma caribbica* as a starter culture: technological applications

Several recent applied studies show that *M. caribbica* can influence the dynamics and quality of fermented products, especially in coffee and related beverages, by altering volatile profiles, suppressing undesirable fungi, and improving sensory attributes (Bernardes et al. 2024). In controlled starters, *M. caribbica* may contribute desirable esters and higher alcohols, modulate acidity through organic–acid production, and act synergistically with lactic acid bacteria to stabilize fermentation trajectories and enhance overall flavor development.

Although *Saccharomyces* and *Kluyveromyces* species dominate dairy starters, incorporating non–*Saccharomyces* yeasts can enrich aroma complexity and enzymatic activity in fermented milks and plant matrices. Careful strain selection for proteolytic activity and lactose/oligosaccharide metabolism is essential, given that dairy matrices are targeted properties that have been emphasized in recent assessments of non-*Saccharomyces* yeast performance (Imre and Crook 2025).

Beyond organoleptic benefits, *M. caribbica* has demonstrated antagonistic mechanisms against fungal pathogens via nutrient competition, VOCs, and hydrolytic enzymes—properties which can reduce spoilage and mycotoxin risk during fermentation and storage (Herrera-Balandrano et al. 2023). This multifunctional profile makes it attractive for starter formulations intended to both improve flavour and enhance safety, making it an appealing candidate for innovative fermented-food formulations.

## Scale–up, formulation, commercialization, and regulatory considerations

*Meyerozyma caribbica* has shown great promise in laboratory and pilot-scale trials, and efforts are underway to transition this yeast from research to commercial application. Several studies have focused on formulating *M. caribbica* into stable products suitable for industrial use on produce. For instance, researchers have developed dry powder and microencapsulated formulations of *M. caribbica* to improve its shelf-life, stress tolerance, and ease of handling (Aguirre-Güitrón et al. 2022). Spray-drying techniques have been optimized to produce *M. caribbica* in a dormant but viable form that can be reactivated when applied to fruits (Aguirre-Güitrón et al. 2018). Microencapsulation in biopolymers (e.g. alginate or pectin matrices) has been especially promising. Encapsulated *M. caribbica* cells are protected from desiccation and UV damage and can be sprayed onto crops or produce surfaces with sustained viability (González-Gutiérrez et al. 2021). Such advances highlight the progress toward practical deployment, moving beyond the lab petri dish to real-world storage and supply chain conditions*. Meyerozyma caribbica* has now been tested on a variety of fruits (mangoes, citrus, apples, pears, berries, among others) and against a broad spectrum of pathogens with consistently positive results (Herrera-Balandrano et al. 2023). Given its wide host range and modes of action, *M. caribbica* could be positioned as a next-generation biocontrol product for postharvest disease management, either as a stand-alone yeast preparation or in combination with other products. These findings are guiding best practices for how a future *M. caribbica*-based product might be applied in packing houses. Overall, the trajectory from bench to market for *M. caribbica* is advancing, supported by formulation technology and promising semi-commercial trials.

Regulatory approval of *M. caribbica* as a commercial biocontrol agent will require careful safety and risk assessments, and this remains a critical gating factor. Unlike well-known biocontrol yeasts such as *Cryptococcus* or *Metschnikowia, M. caribbica* belongs to a yeast clade (the *Meyerozyma*/*Candida guilliermondii* species complex) that includes opportunistic human pathogens. Indeed, some strains of *M. guilliermondii* are documented causes of candidemia and other infections in immunocompromised patients (Chen et al. 2020). A multicenter survey across Latin America reported that *M. caribbica* accounted for 3.4% of candidemia cases between 2000 and 2008, rising to 13.8% during 2009–2018 (Francisco et al. 2023). This means regulators will scrutinize *M. caribbica*’s safety profile to ensure it does not pose a risk to handlers, consumers, or the environment.

Sibirny and Boretsky (2009) affirm that during decades of work with this species, no cases of candidiasis were caused by their laboratory strains of *M. guilliermondii*. The authors recognize the yeast type strains as Generally Recognized as Safe (GRAS). However, it is important to be cautious about this classification. In fact, Corte et al. (2015) stated that *M. guilliermondii* should have a questionable GRAS or Qualified Presumption of Safety (QPS) status, concluding that this species is neither a QPS nor a non-QPS classified micro-organism under European legislation For these reasons, formal registration of *M. caribbica* as a biopesticide will require regulatory agencies to review comprehensive safety data. Such data typically include acute toxicity tests in animal models that show no harm. However, toxicity assessments for the reported biocontrol strains of *M. caribbica* are lacking. Therefore, their potential toxic effects remain uncertain, representing a significant knowledge gap that should be addressed in future research. Toxicity evaluation should be a prerequisite for the commercial application of these strains. Nevertheless, it is noteworthy that *M. guilliermondii* and *M. caribbica* do not produce toxic metabolites, which constitutes a clear advantage over many other fungal taxa (Herrera-Balandrano et al. 2023).

Besides its applications in biocontrol, some strains of *M. caribbica* have also been evaluated in food and fermentation contexts, where technological and regulatory requirements differ from those applied to biopesticides. Robust production workflows, including controlled fed-batch cultivation and careful harvest to preserve cell wall integrity, are necessary for viable starter or probiotic products. Downstream options include frozen concentrates, dried powders obtained by spray- or freeze-drying with protective carriers, or live liquid starters, requiring optimization to retain viability and functional traits (Moonsamy et al. 2024). Starter cultures for food production must be characterized for stability, genetic drift, and batch–to–batch consistency. Strain bank management and standard operating procedures for propagation reduce phenotypic variability that could affect sensory outcomes (de Marco et al. 2018).

Regulatory acceptance for probiotic yeasts and starter cultures varies regionally. While *S. cerevisiae* var. *boulardii* and some *S. cerevisiae* strains enjoy established use, non–*Saccharomyces* candidates typically need strain–level safety dossiers. Whole Genome Sequencing (WGS), absence of virulence or transferable resistance determinants, and documented absence of harmful metabolites form the backbone of regulatory submissions (Wang et al. 2021). Current evidence for *M. caribbica* is encouraging but still incomplete. Most reports are *in vitro* or small–scale fermentations, and long–term safety and human clinical data for this yeast remain limited. Comparative multi–omics (metabolomics + genomics) in consortia of *M. caribbica* with lactic acid bacteria and *Saccharomyces* in pilot–scale fermentations would clarify mechanisms underpinning sensory and functional outcomes (Bernardes et al. 2024, Moonsamy et al. 2024).

## Concluding remarks

**Figure 4:**
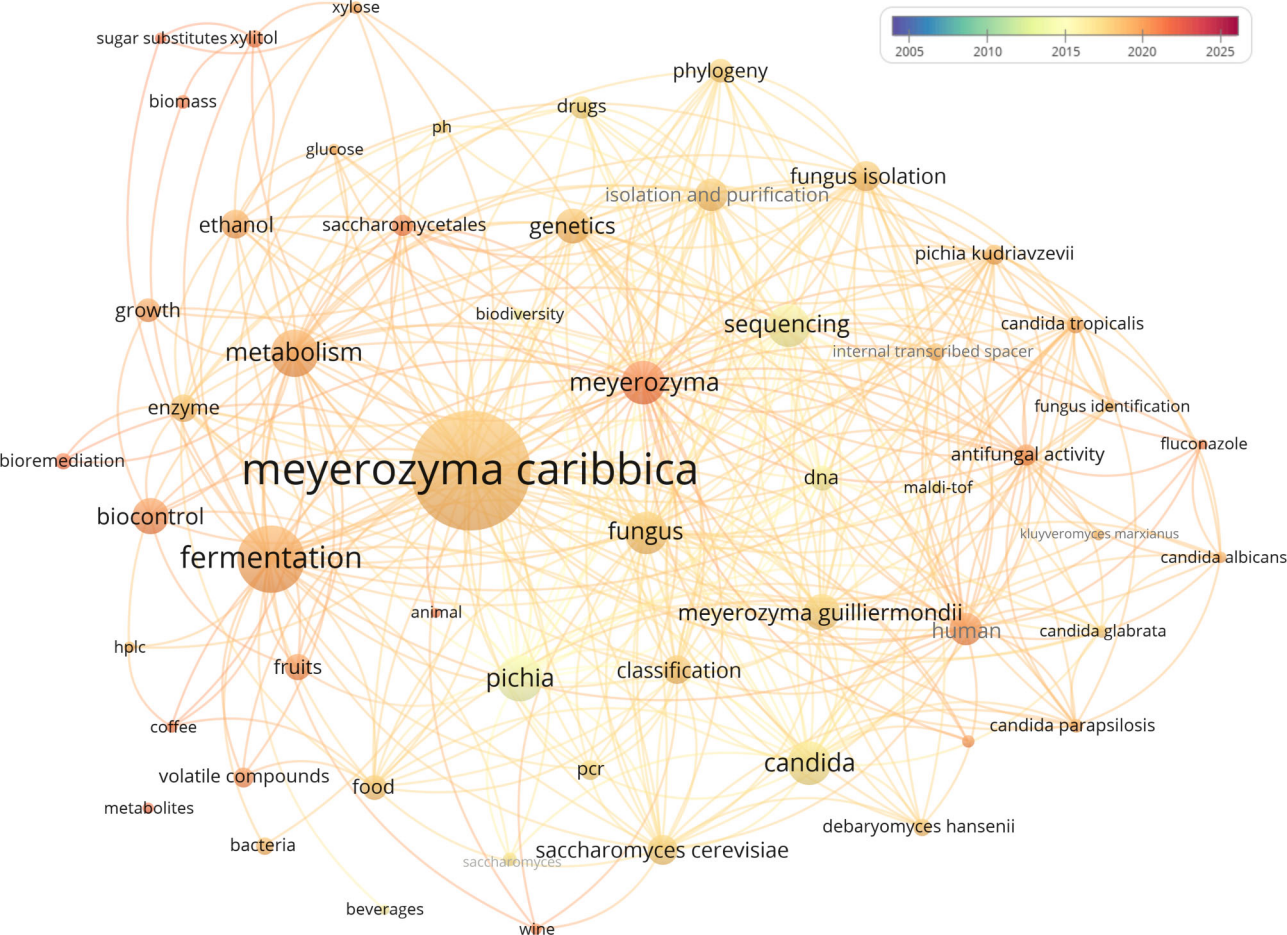
Bibliometric co-occurrence map of keywords associated with *M. caribbica*. The dataset consisted of 232 references retrieved from Scopus using the following search query: TITLE-ABS-KEY (*caribbica*) AND [LIMIT-TO (DOCTYPE, “ar”)]. The co-occurrence map was generated in VOSviewer, after harmonizing different keywords with equivalent meanings. In total, the 232 references yielded 2723 unique keywords, from which only those with a minimum of 10 occurrences were included in the analysis. For visualization clarity, the map displays the 400 strongest correlation links among the selected terms. The color scale represents the average publication count for each term over the analyzed twenty-year timespan.

Over the past two decades, *M. caribbica* has gradually transitioned from an understudied yeast species to a promising biological agent in multiple areas of applied microbiology. As scientific interest grew, so did the diversity of reported applications, particularly in contexts where metabolic versatility and ecological adaptability are required. This expanding research landscape is effectively captured in the bibliometric map generated for this review (Fig. 4). The map highlights how, among biotechnological applications, the most prominent terms are “fermentation”, “metabolism”, and “biocontrol”. While the association with the words fermentation and metabolism is expected for a yeast species, the strong emergence of “biocontrol”—accompanied by related terms such as “fruits,” “food,” and “volatile compounds”—reinforces the central role of *M. caribbica* in postharvest disease management and food-related contexts. Our comprehensive survey demonstrates that recent research increasingly highlights the species' efficacy in suppressing fungal pathogens and modulating volatile compound profiles, dual attributes that underpin its expanding application as a biological control agent.

In this context, the presence of “fungus,” “antifungal activity”, and “fungus isolation” among the frequent keywords reflects the biological underpinnings of its biocontrol potential. These patterns align with the evidence reviewed here, which demonstrates how the species produces metabolites and enzymes capable of suppressing competing fungi. Considering the global demand for sustainable, low-impact agricultural technologies, such antifungal activity positions *M. caribbica* as a valuable candidate for biological alternatives to chemical fungicides, with implications for both conventional and organic production systems. Its multifunctional traits (such as inhibition of plant pathogens, environmental resilience, and metabolic flexibility) strengthen its relevance in developing environmentally responsible strategies for crop protection and food production.

Finally, the strongest keyword signals correspond to publications from the most recent years, particularly after ∼2020, indicating a marked acceleration of research activity. This temporal pattern suggests that *M. caribbica* is gaining visibility as a versatile platform organism with multiple avenues for applied innovation. Given this trajectory and the substantial accumulation of knowledge synthesized in this review, *M. caribbica* stands poised to become an increasingly important yeast species in biotechnology, agriculture, environmental applications, and food sciences. This consolidated understanding provides a foundation for future experimental work, encourages systematic exploration of its metabolic and ecological properties, and highlights its rising importance as a non-conventional yeast with far-reaching potential.

## Supplementary Material

foag010_Supplemental_Files
